# The success rate of small renal mass core needle biopsy and its impact on lowering benign resection rate

**DOI:** 10.1186/s12894-023-01363-x

**Published:** 2023-11-18

**Authors:** Haijuan Gao, Behdokht Nowroozizadeh, Joaquin Ponce Zepeda, Jaime Landman, Ted Farzaneh, Cary Johnson, Hirad Hosseini, Min Han

**Affiliations:** 1 Department of Pathology and Laboratory Medicine, University of California, Irvine, Orange, CA USA; 2 Department of Urology, University of California, Irvine, Orange, CA USA; 3 University of Southern California, Los Angeles, CA USA; 4 Department of Pathology, City of Hope Medical Center, 1500 E. Duarte Road, Duarte, CA 91010 USA

**Keywords:** Renal Mass, Renal cell carcinoma, Core Needle Biopsy, Resection

## Abstract

**Background:**

Small renal mass (SRM) biopsy remains under-utilized due to stigma. Meanwhile, the alarmingly high benign findings in resected kidney masses highlight the need for improved preoperative diagnosis and patient selection.

**Methods:**

The purpose of this study is to review the success rate of SRM biopsy and to evaluate its impact on patient management. A total of 168 percutaneous image-guided core needle biopsies (CNBs) of SRMs were retrieved at a tertiary academic center between 2015 and 2019. Subsequent treatment choices, side effects and outcomes were retrospectively reviewed.

**Results:**

The diagnostic rate of CNB was 86.9%. Benign neoplasms accounted for a significant portion (14.3%) of SRM. Renal cell carcinomas (RCCs) were the most common diagnoses (69.6%) as expected. In biopsy-resection correlation, the positive predictive value of CNB was 100%. Tumor typing and subtyping by CNB were highly accurate, 100% and 98.3% respectively. Nuclear grading for clear cell RCC was accurate in 83.8% cases. The CNB results had significant impact on treatment. Most patients with RCCs underwent either resection (54.1%) or ablation (33.9%), in contrast to observation in benign neoplasms (90.5%). Most importantly, the benign resection rate (3.2%) in this series was much lower than the national average.

**Conclusion:**

CNB provided accurate diagnoses for the majority of SRMs and revealed benign diagnoses in a subset of clinically suspicious lesions. Employment of CNB in suspicious SRM may help avoid overtreatment for benign lesions.

## Background

Solid renal masses represent a diverse group of conditions [1–3]. Most commonly, renal cell carcinoma (RCC) is the predominant type of kidney cancer. Other mass-forming lesions in the kidney include benign neoplasms, such as oncocytoma, angiomyolipoma and papillary adenoma. Urothelial carcinoma, metastatic carcinoma, lymphoma and inflammatory processes account for a small percentage of solid renal masses. Management of solid renal masses depend on the diagnosis. While surgery or ablation are often employed for patients with RCCs, benign and indolent tumors may be followed with active surveillance [4, 5]. Meanwhile, metastatic carcinomas and lymphomas often require chemotherapy.

The incidence of kidney cancer has steadily increased from 10 to 15.4 per 100,000 persons between 1992 and 2019 [6] (https://seer.cancer.gov/statfacts/html/kidrp.html). The greatest increase is in small renal masses (SRMs) defined as localized renal masses measuring 4 cm or less in greatest dimension(cT1a) [7, 8]. This is mostly due to increased detection of SRMs by computed tomography (CT) and magnetic resonance imaging (MRI) [2]. SRMs have represented up to 40% of the incidental tumors in multiple studies [9, 10]. Traditionally, treatment of suspicious renal masses has been relying on imaging diagnosis. To date, small biopsies including core needle biopsy (CNB) and fine needle aspiration (FNA) are not required before surgery. This is in contrast to most other organ systems which usually require pathologic diagnosis prior to definitive therapy.

Paradoxically, increased detection of SRM has not been associated with significant improvement of patient outcome. Instead, overtreatment of benign renal masses became an emerging issue. A large database analyzation revealed that over 30% of partial nephrectomy specimens contained benign findings only [11].

Renal mass core needle biopsy has been brought into discussion as part of the algorithm to manage solid renal masses [12–18]. CNB can distinguish benign from malignant lesions, and to rule out metastasis, hematologic and inflammatory processes. Diagnosis of benign renal masses by CNB has provided assurance for the non-surgical treatment. This is pertinent more than ever before duo to an aging population and the need to preserve kidney functions [4, 5]. Last but not least, CNB can also acquire tissue for molecular testing for targeted therapies in large primary tumors and in metastatic settings [14]. Despite of all the benefits, renal mass CNB has not been widely adopted to date due to the perceived low yield and concern for complications. The reported biopsy rate in modern literature was only 7–15% [14, 19]. A recent survey among 1,131 responding urologists practicing in the U.S. showed that 32% of them would never biopsy a renal mass less than 4 cm [20]. The purpose of this study was to demonstrate the merit of CNB in the management of small renal masses by reviewing the accuracy of CNB in diagnosing clinically suspicious small renal masses and its impact on treatment.

## Methods

### Study design

Institutional review board approval (IRB Number: HS# 2019–5602) was obtained from University of California Irvine for a retrospective case series review of renal mass CNBs. All renal mass CNBs (in total of 265) performed between January 2015 and December 2019 were retrieved from the University of California Irvine Medical Center database. Only small renal masses measuring 4 cm or less in greatest dimension were included in the final analysis.

The medical records of 159 patients with 168 CNBs were retrospectively reviewed. Patient’s demographic characteristics such as age, gender and both biopsy and resection histopathological result and radiographic characteristics, biopsy related complications, subsequent treatment and follow up information were recorded from electronic medical record.

It is the policy of the surgeon (JL) involved in the management of the current cohort of patients to routinely offer renal biopsy to all cT1a (< 4 cm) patients. The risks and benefits of biopsy are discussed for shared decision making. In this process, it is very rare for patients to defer biopsy. As such, it is routine for the vast majority of patients to undergo biopsy prior to establishing a definitive management strategy.

### Biopsy technique

Per medical record, moderate sedation was achieved during the procedure using benzodiazepines and opioids. In addition, local anesthesia (1% lidocaine) was applied to the skin at the biopsy site. Most biopsies were performed using 17-gauge coaxial introducer needle and 18- to 20-gauge Temno biopsy needle. As routine practice in the institution, rapid on-site evaluation (ROSE) by cytopathologists either in person or using tele-cytopathology was performed using Diff-Quik stained touch-preparation (TP) slides for all CNBs, and adequacy of the samples were recorded during ROSE. H&E-stained slides from formalin-fixed paraffin-embedded (FFPE) tissue blocks and immunohistochemistry were used for final diagnosis. CNB and resection diagnoses, tumor types and subtypes, World Health Organization (WHO)/International Society of Urologic Pathologists (ISUP) nuclear grade for clear cell renal cell carcinomas (RCC) and number of needle cores acquired were recorded from the pathology reports.

CNB diagnoses were correlated with resection diagnoses and clinical/radiologic findings. Cases with discrepancies between CNB and resection diagnoses were separately reviewed by three surgical pathologists (M.H, T. F and C. J), blind of the original diagnoses. The estimated percentage of tumor with low grade (G1-G2) nuclei and high grade (G3-G4) nuclei were provided for each case and compared among the three pathologists, with an additional review of the original diagnosis. Inter-observer variability was recorded when a different nuclear grade was given to the same case by the pathologists.

### Statistical analysis

Descriptive statistical were used for demographic, pathological and clinical data. Statistical analysis was performed using IBM SPSS Statistics 28.0.

## Results

265 consecutive renal mass biopsies were identified between January 2015 and December 2019. After exclusion of 97 cases (exclusion criteria: tumor size greater than 4 cm or size unknow in 94 cases and samples acquired by FNA in 3 cases), 168 SRM CNBs were included in the final analysis. Nine of the 168 cases were biopsied twice due to initial negative (n = 8) or indeterminate atypical (n = 1) results. Patients’ demographics and tumor characteristics were shown in Table 1. Briefly, all renal masses were 4 cm or smaller in size and were solid or solid-cystic with imaging features suspicion for renal cell carcinomas. Percutaneous CNBs were performed by experienced urologists or interventional radiologists under CT and/or ultrasound guidance. Complications such as hemorrhage, severe pain, hematuria and infection occurred in rare events (Table 1).

As a result, 82.7% (139/168) of SRMs achieved specific histologic diagnosis by the initial CNB. After repeating biopsy in selected cases (n = 9), the diagnostic rate increased to 86.9% (Table 2). The final diagnoses included renal cell carcinomas (RCCs) (69.9%), benign renal neoplasms (14.3%) and other types of malignancy other than RCC (2 metastatic carcinomas, 1 lymphoma and 1 urothelial carcinoma).

**Table 1:** Demographic and tumor characteristics (N = 168)

Characteristics	Sub-categories	No. (%)	
Age (yrs)	Mean = 63.4 yrs	Range (28, 87)	
Gender	Male	103	(61.7)
	Female	65	(38.3)
Laterality	Left	82	(49.1)
	Right	85	(50.9)
Tumor Size (Imaging)	1–2 cm	45	(26.9)
	2.1-4 cm	123	(73.1)
Internal structure	Solid	90	(53.3)
	Solid cystic	9	(5.4)
	Unknown	69	(41.3)
Enhancement	Enhancing	98	(57.5)
	Non-enhancing	4	(2.4)
	Unknown	66	(39.6)
Modality	US-guided	48	(28.6)
	CT-guided	119	(70.8)
	US + CT	1	(0.6)
Number of Cores	1–2	47	(28.1)
	3–4	93	(55.1)
	5–6	22	(13.2)
	7 and more	5	(3.0)
Side effect	Hemorrhage	5	(3.0)
	Tumor Seeding	0	(0.0)
	Severe pain	3	(1.8)
	Infection	1	(0.6)
	Hematuria	3	(1.8)
Treatment	Surgery	63	(37.5)
	Ablation	41	(24.4)
	Surveillance	50	(29.8)
	Chemotherapy	2	(1.2)
	No follow up	12	(7.1)

**Table 2:** Histologic diagnoses of 168 renal masses based on subcutaneous image-guided core needle biopsies (CNB)

CNB Diagnosis	Subtypes	Initial CNB No. (%)		After 2nd CNB* No. (%)	
RCC		112	(66.7)	117	(69.6)
	Clear Cell	69	(41.1)	74	(44.0)
	Papillary	19	(11.3)	19	(11.3)
	Chromophobe	9	(5.4)	9	(5.4)
	Rare variant	5	(3.0)	5	(3.0)
	Un-subclassified	10	(6.0)	10	(6.0)
Benign Neoplasm		22	(13.1)	24	(14.3)
	Oncocytoma	16	(9.5)	18	(10.7)
	Angiomyolipoma	5	(3.0)	5	(3.0)
	Papillary Adenoma	1	(0.60)	1	(0.60)
Malignant, non-RCC		5	(3.0)	5	(3.0)
***Subtotal***		***139***	***(82.7%)***	***146***	***(86.9%)***
Atypical		7	(4.2)	6	(3.6)
Negative		22	(13.1)	16	(9.5)
***Total***		***168***	***(100.0)***	***168***	***(100.0)***

* Nine patients underwent repeat CNB for negative (n = 8) or atypical (n = 1) diagnosis by initial CNB

Abbreviations: CNB, core needle biopsy; RCC, renal cell carcinoma; UMP, unknown malignant potential; AML, Angiomyolipoma

The most common benign findings were oncocytomas (n = 18, 10.7%), followed by angiomyolipoma (n = 5, 3.0%) and papillary adenoma (n = 1, 0.6%) in the current study.

Only 3.6% of CNBs were indeterminate due to scant atypical cells or low grade oncocytic neoplasms (classified as atypical in the following text). In addition, 9.5% CNBs contained only non-neoplastic renal parenchyma or fibroadipose tissue. These negative and atypical diagnoses were problematic. Particularly, the 9.5% CNBs with only non-neoplastic tissue may represent true negativity or false negativity. Further clinical investigation was needed in these cases.

Following CNB diagnosis, partial or total nephrectomies were documented in 66 (39.3%) cases. The time period from CNB to resection was 3.4 months (range: 0.5 to 21 months). 63 cases had resection slides and/or pathology reports available for correlation with CNB diagnoses (Table 3). After reviewing the CNB and resection slides and/or pathology reports, pathologists confirmed that all malignant diagnoses made by CNB were concordant with final diagnosis, therefore, the positive predictive value of CNB was 100%.

**Table 3:** Concordance of tumor types and subtypes between biopsy and resection diagnoses in resected renal masses (total N = 63)

CNB Diagnosis			N	Resection Diagnosis		N	Concordance Rate (%)	
RCC			58	RCC		58	100	
	*Clear cell*		*41*		*Clear cell*	*40*	*97.6*	*(subtypes)*
					*Chromophobe*	*1*		
	*Papillary*		*7*		*Papillary*	*7*	*100*	*(subtype)*
	*Chromophobe*		*4*		*Chromophobe*	*4*	*100*	*(subtype)*
	*Rare variant*		*2*		*Rare variant*	*2*	*100*	*(subtype)*
	*Un-subclassified*		*4*		*Clear Cell*	*4*	*0*	*(subtype)*
Oncocytoma			1	Oncocytoma		1	100	
Atypical			1	Clear cell RCC		1	0	
Negative			3	Clear cell RCC		1	0	
				Papillary RCC		1		
				AML		1		

Abbreviations: CNB, core needle biopsy; RCC, renal cell carcinoma; UMP, unknown malignant potential; AML, Angiomyolipoma; CCRCC, Clear cell renal cell carcinoma

To calculate the negative predictive value, all or most negative CNBs should have gold standard (resection) diagnosis for correlation, which is not realistic in real practice. In this series, only three of the 16 CNB-negative cases were resected for gold standard diagnosis, making it challenging to know the true negative predictive value. Based on the resection diagnoses (2 RCCs and 1 angiomyolipoma) in these three CNB-negative cases, the false negative rate was at least 12.5% (2/16).

Benign resection rate of SRMs was an important indicator for overtreatment. In this series, two of the 63 resected SRMs were benign (1 oncocytoma, 1 angiomyolipoma). Therefore, the benign resection rate was 3.2%. Interestingly, the oncocytoma was correctly diagnosed on CNB prior to resection. The angiomyolipoma (AML) case was missed in the CNBs due to sampling error.

CNB also provided accurate subclassification and nuclear grading for RCCs. Tumor subtypes were provided in 93.1% (54/58) of RCC cases in CNB, and the results were concordant with the final tumor subtypes in 98.3% (58/58) cases (Table 3). WHO/IUSP (formerly Fuhrman) nuclear grade was reported in 37 clear cell RCCs in CNBs. The results were concordant with the final nuclear grade in 83.8% (31/37) cases.

**Fig. 1:**
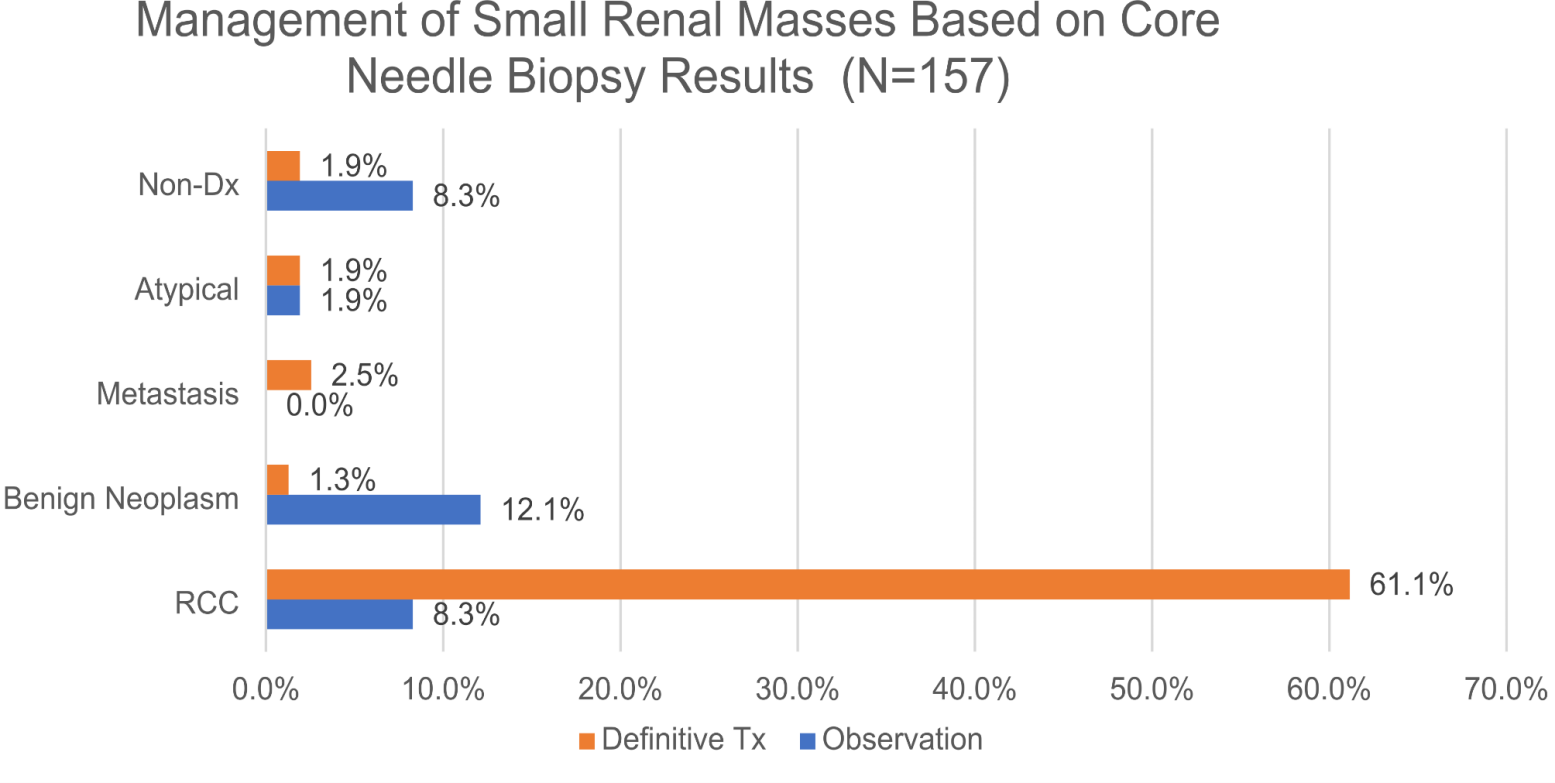
The main treatment choices for RCC were resection and ablation (definitive treatment). In contrast, the majority of patients with benign neoplastic diagnoses underwent clinical observation with or without radiological follow ups. Chemotherapy (definitive treatment) was applied in few patients with other malignancies such as metastatic carcinoma and lymphoma.( Abbreviations: RCC – renal cell carcinoma; Non-Dx – non-diagnostic; Definitive Tx – Definitive treatment.)

Follow-up treatment information was available for 157 (93.5%) patients (Fig. 1). The main treatment choices for RCC were resection (54.1%) or ablation (33.9%). Together these definitive treatments were applied to 88.1% of RCC patients. In contrast, the majority (90.5%) of patients with benign neoplastic diagnoses underwent clinical observation with or without radiological follow ups. Lastly, chemotherapy was chosen for two patients with metastatic carcinoma and lymphoma respectively. In subsequent clinical and radiologic follow-ups, no adverse events were reported associated with benign neoplasms diagnosed by CNB.

## Discussion

This study supported that CNB was high yield in diagnosing SRMs. CNBs achieved specific histologic diagnosis in 86.9% (146/168) of SRMs in this study, similar to previous reports (80 − 95%) [3, 15]. CNBs not only provided histologic evidence of malignancy versus benignity, but also provided prognostic information such as tumor subtypes and nuclear grading. The accuracy of tumor typing (100%), subtyping (97.3 -100%) and nuclear grading for CCRCC (83.8%) in this study was similar to data from other groups [15, 16, 21–23].

Core needle biopsy had a great impact on the outcomes of this cohort. A significant number of patients (24/168, 14.3%) with suspicious small renal masses were reassured of benign diagnosis by core needle biopsies. As a result, the great majority (over 90%) of these patients chose active surveillance instead of partial nephrectomy or ablation, which would have been recommended to these patients at other institutions where CNB is not routinely performed. Clearly, biopsy allows for incorporation of histopathology into the decision-making process. As such, the benign resection rate has been reduced to a minimal level (3.2%) at our institution compared to national average (over 30%) [11, 24]. A systemic review of surgical series and United States population level burden estimate found that benign histology accounted for 40.4% of resected renal masses measuring less than 1 cm, and that misclassified benign lesions remained high (17.2%) for renal masses measuring 3–4 cm [9]. On the other hand, this study demonstrated that routine application of biopsy into the management of small renal masses was transformative and almost completely eliminated benign pathology on surgery. Similarly, other studies have also shown renal tumor biopsy reduces surgery for benign tumors [24, 25]. An analysis of 106,258 patients with small renal masses from the National Cancer Data Base (NCDB) from 2004 to 2015 showed increased use of renal biopsy (from 8.0 to 15.3%) and an associated increase in non-surgical management (from 11.7 to 15.6%). Altogether, these data suggest that SRM CNB has the potential to alter patient management and reduce the risk of overtreatment and mistreatment. In addition, a small number of patients were confirmed to have metastatic carcinomas or lymphoma, for which systemic chemotherapy was given rather than surgery or ablation. Therefore, CNBs should be considered before invasive interventions for any SRMs.

A common concern, however, for renal mass biopsy is the non-diagnostic results [23, 26]. Based on a previous review of 20 studies including 2,979 patients and 3,113 biopsies of localized renal masses, the non-diagnostic rate of renal mass core biopsy was as high as 13.9%, among which, 90.4% were diagnosed as malignant lesions upon resection [17]. In the current study, among the 16 negative/non-diagnostic CNBs, 2 cases proved to be malignant in resection, leading to a false negative rate of at least 12.5%. Another patient underwent partial nephrectomy for AML due to non-diagnostic CNB result. These results underscore the importance of clinical and radiological correlation when the biopsy is clearly non-diagnostic or yields only normal kidney parenchyma. To improve the diagnostic rate, repeat biopsy may be considered in patients with high clinical suspicion [15, 22, 23]. In addition, rapid onsite evaluation by touch preparation may have some merit in improving the diagnostic yield of small biopsies.

It’s also noted that nuclear grading by CNB was less than optimal given that 16.2% of CCRCCs were eventually upgraded to higher nuclear grade on resection. The discrepancy of nuclear grade was mostly due to intra tumoral heterogeneity as previously discussed [1, 27–29]. Therefore, cautions should be exercised when determine the treatment options to avoid undertreatment of focally aggressive tumors.

Overall, CNB diagnosis had a significant impact on the treatment for patients with small renal masses. Most RCCs were managed by resection or ablation while surgery was rarely applied to benign lesions and non-RCC type malignant tumors. Additionally, the management of the atypical and negative/non-diagnostic cases was variable, which in turn highlighted the challenge for managing patients without clear pathologic diagnosis.

The limitation of this study is the relatively small size of the cohort. Also, the retrospective chart review may not capture all the nuances and factors that might also have contributed to the management decisions for all patients. Additional studies focusing on radiological-pathological correlation may provide insight on how to further stratify malignant risks based on radiologic features.

## Conclusions

This study provides evidence that CNB is high yield in diagnosing renal mass lesions. The majority (86.9%) of biopsied lesions achieved histopathological diagnosis. Repeat biopsy can help lower the negative results. Tumor types and subtypes diagnosed by CNB were highly concordant with resection diagnoses. Furthermore, the most important messages from this study, is that the CNB results were shown to affect patient treatment plans. The use of RMB can help avoid overtreatment of benign renal lesions.

## Data Availability

The datasets used and/or analyzed during the current study are available from the corresponding author on reasonable request.

## Abbreviations

SRM: Small renal mass
RCC: Renal cell carcinoma
CNB: Core needle biopsy
CT: Computed tomography
MRI: Magnetic resonance imaging
CCC: Clear cell carcinoma
FNA: Fine needle aspiration
ROSE: Rapid on-site evaluation
NCDB: National Cancer Data Base
FFPE: Formalin-fixed paraffin-embedded
AML: Angiomyolipoma

## References

[1] Beksac AT, Paulucci DJ, Blum KA, Yadav SS, Sfakianos JP, Badani KK (2017). Heterogeneity in renal cell carcinoma. Urol Oncol.

[2] Kay FU, Pedrosa I (2018). Imaging of solid renal masses. Urol Clin North Am.

[3] Tomaszewski JJ, Uzzo RG, Smaldone MC (2014). Heterogeneity and renal mass biopsy: a review of its role and reliability. Cancer Biol Med.

[4] Cheung DC, Finelli A (2017). Active surveillance in small renal masses in the Elderly: A literature review. Eur Urol Focus.

[5] Jewett MA, Mattar K, Basiuk J, Morash CG, Pautler SE, Siemens DR, Finelli A (2011). Active surveillance of small renal masses: progression patterns of early stage kidney cancer. Eur Urol.

[6] Ljungberg B, Campbell SC, Choi HY, Jacqmin D, Lee JE, Weikert S, Kiemeney LA (2011). The epidemiology of renal cell carcinoma. Eur Urol.

[7] Almassi N, Gill BC, Rini B, Fareed K (2017). Management of the small renal mass. Transl Androl Urol.

[8] Jayson M, Sanders H (1998). Increased incidence of serendipitously discovered renal cell carcinoma. Urology.

[9] Johnson DC, Vukina J, Smith AB, Meyer AM, Wheeler SB, Kuo TM, Nielsen ME (2015). Preoperatively misclassified, surgically removed benign renal masses: a systematic review of surgical series and United States population level burden estimate. J Urol.

[10] Nguyen MM, Gill IS, Ellison LM. (2006). The evolving presentation of renal carcinoma in the United States: trends from the Surveillance, Epidemiology, and End Results program. *J Urol, 176*(6 Pt 1), 2397–2400; discussion 2400. 10.1016/j.juro.2006.07.144. 10.1016/j.juro.2006.07.144 17085111

[11] Kim JH, Li S, Khandwala Y, Chung KJ, Park HK, Chung BI (2019). Association of Prevalence of Benign pathologic findings after partial nephrectomy with preoperative imaging patterns in the United States from 2007 to 2014. JAMA Surg.

[12] Alle N, Tan N, Huss J, Huang J, Pantuck A, Raman SS (2018). Percutaneous image-guided core biopsy of solid renal masses: analysis of safety, efficacy, pathologic interpretation, and clinical significance. Abdom Radiol (NY).

[13] Halverson SJ, Kunju LP, Bhalla R, Gadzinski AJ, Alderman M, Miller DC, Wolf JS (2013). Accuracy of determining small renal mass management with risk stratified biopsies: confirmation by final pathology. J Urol.

[14] Herrera-Caceres JO, Finelli A, Jewett MAS (2019). Renal Tumor biopsy: indicators, technique, safety, accuracy results, and impact on treatment decision management. World J Urol.

[15] Jeon HG, Seo SI, Jeong BC, Jeon SS, Lee HM, Choi HY, Jeong IG (2016). Percutaneous kidney biopsy for a small renal Mass: a critical Appraisal of results. J Urol.

[16] Neuzillet Y, Lechevallier E, Andre M, Daniel L, Coulange C (2004). Accuracy and clinical role of fine needle percutaneous biopsy with computerized tomography guidance of small (less than 4.0 cm) renal masses. J Urol.

[17] Patel HD, Johnson MH, Pierorazio PM, Sozio SM, Sharma R, Iyoha E, Allaf ME (2016). Diagnostic accuracy and risks of Biopsy in the diagnosis of a renal Mass Suspicious for localized renal cell carcinoma: systematic review of the literature. J Urol.

[18] Pierorazio PM, Johnson MH, Patel HD, Sozio SM, Sharma R, Iyoha E, Allaf ME. (2016). In *Management of Renal Masses and Localized Renal Cancer*. Rockville (MD). 27010048

[19] Richard PO, Martin L, Lavallee LT, Violette PD, Komisarenko M, Evans AJ, Finelli A (2018). Identifying the use and barriers to the adoption of renal tumour biopsy in the management of small renal masses. Can Urol Assoc J.

[20] Patel RM, Safiullah S, Okhunov Z, Meller D, Osann K, Kaler K, Clayman RV (2018). Pretreatment diagnosis of the small renal Mass: Status of Renal Biopsy in the United States of America. J Endourol.

[21] Richard PO, Jewett MA, Bhatt JR, Kachura JR, Evans AJ, Zlotta AR, Finelli A (2015). Renal Tumor biopsy for small renal masses: a single-center 13-year experience. Eur Urol.

[22] Richard PO, Jewett MA, Tanguay S, Saarela O, Liu ZA, Pouliot F, Finelli A (2017). Safety, reliability and accuracy of small renal tumour biopsies: results from a multi-institution registry. BJU Int.

[23] Sutherland EL, Choromanska A, Al-Katib S, Coffey M (2018). Outcomes of ultrasound guided renal mass biopsies. J Ultrasound.

[24] Richard PO, Lavallee LT, Pouliot F, Komisarenko M, Martin L, Lattouf JB, Finelli A (2018). Is routine renal Tumor Biopsy Associated with Lower Rates of Benign Histology following nephrectomy for small renal masses?. J Urol.

[25] Patel HD, Nichols PE, Su ZT, Gupta M, Cheaib JG, Allaf ME, Pierorazio PM (2020). Renal Mass Biopsy is Associated with reduction in Surgery for early-stage kidney Cancer. Urology.

[26] Patel HD, Pierorazio PM (2016). Kidney cancer: undertreatment of small renal masses by overuse of biopsy. Nat Rev Urol.

[27] Hsieh JJ, Le V, Cao D, Cheng EH, Creighton CJ (2018). Genomic classifications of renal cell carcinoma: a critical step towards the future application of personalized kidney cancer care with pan-omics precision. J Pathol.

[28] Lopez JI (2016). Intratumor heterogeneity in clear cell renal cell carcinoma: a review for the practicing pathologist. APMIS.

[29] Lopez JI, Angulo JC (2018). Pathological bases and clinical impact of Intratumor Heterogeneity in Clear Cell Renal Cell Carcinoma. Curr Urol Rep.

